# The Genome Stability Maintenance DNA Helicase DDX11 and Its Role in Cancer

**DOI:** 10.3390/genes12030395

**Published:** 2021-03-10

**Authors:** Mohammad Mahtab, Ana Boavida, Diana Santos, Francesca M. Pisani

**Affiliations:** 1 Istituto di Biochimica e Biologia Cellulare—CNR, Via P. Castellino, 111, 80131 Naples, Italy; md.mahtab@ibbc.cnr.it (M.M.); ana.boavida@ibbc.cnr.it (A.B.); diana.santos@ibbc.cnr.it (D.S.); 2 Dipartimento di Scienze e Tecnologie Ambientali Biologiche e Farmaceutiche, Università degli Studi della Campania Luigi Vanvitelli, Via Vivaldi, 43, 81100 Caserta, Italy

**Keywords:** DDX11/ChlR1, genome stability, sister chromatid cohesion, DNA helicase, long noncoding RNAs, human papillomavirus, cancer, oncogene, tumor suppressor gene

## Abstract

DDX11/ChlR1 is a super-family two iron–sulfur cluster containing DNA helicase with roles in DNA replication and sister chromatid cohesion establishment, and general chromosome architecture. Bi-allelic mutations of the DDX11 gene cause a rare hereditary disease, named Warsaw breakage syndrome, characterized by a complex spectrum of clinical manifestations (pre- and post-natal growth defects, microcephaly, intellectual disability, heart anomalies and sister chromatid cohesion loss at cellular level) in accordance with the multifaceted, not yet fully understood, physiological functions of this DNA helicase. In the last few years, a possible role of DDX11 in the onset and progression of many cancers is emerging. Herein we summarize the results of recent studies, carried out either in tumoral cell lines or in xenograft cancer mouse models, suggesting that DDX11 may have an oncogenic role. The potential of DDX11 DNA helicase as a pharmacological target for novel anti-cancer therapeutic interventions, as inferred from these latest developments, is also discussed.

## 1. Introduction

DNA helicases are enzymes able to unwind DNA duplexes and translocate along nucleic acid strands with a specific directionality (3′ to 5′ or vice versa) by utilizing the energy derived from nucleoside triphosphate hydrolysis (typically ATP) [1,2]. Human genome codes for at least 31 different DNA helicases are classified into two major (superfamily 1 and 2) and four minor groups based on their sequence similarity. They are ubiquitous enzymes, found in all kingdoms of life, including bacteriophages and DNA eukaryotic viruses. DNA helicases display different reaction requirements and substrate specificity, being able to melt not only B-form DNA duplex molecules, but also nonconventional DNA structures such as G-quadruplexes and triplexes, displacement loops, Holliday junctions, stem-loops, and cruciform structures. Moreover, some DNA helicases are able to actively displace DNA-bound proteins. DNA helicases play key functions in a variety of DNA replication/repair/recombination pathways that are important for genome stability maintenance. It is, therefore, not surprising that mutations and/or dysregulated expressions of DNA helicase encoding genes are linked to many hereditary diseases, neurodegenerative disorders, and cancers [3]. However, in several instances, the molecular mechanisms underlying the related clinical phenotypes are not fully understood.

The role played by DNA helicases in cancer is complex and depends on the genetic context and the tumor type. In fact, inactivating mutations of DNA helicase genes can predispose to cancer, suggesting a tumor suppressor function for some of these enzymes. Conversely, the level of expression of many DNA helicases is enhanced in pre-cancerous and cancerous cells and tissues compared to the healthy counterparts of the same individual, indicating a pro-tumorigenic behavior of the corresponding enzymes. In this regard, it is well established that certain DNA helicases may help highly proliferating cancer cells to counteract the replication stress deriving from the conflicts between the replication and transcription machineries, and to repair DNA lesions due to either endogenous or exogenous insults [4,5,6].

In this review, we summarize what is known about a potential function in cancer of human DDX11, a DNA helicase, involved in various genome maintenance pathways and in sister chromatid cohesion establishment. We start by providing a general overview of the molecular and cellular roles of DDX11; then, we describe the regulation of DDX11 activity by the long noncoding RNA named *CONCR* and the mechanism by which DDX11 promotes human papillomavirus genome replication and maintenance; a final section is dedicated to the role DDX11 is believed to play in various cancer types.

## 2. Molecular Properties of DDX11 

**Figure 1:**
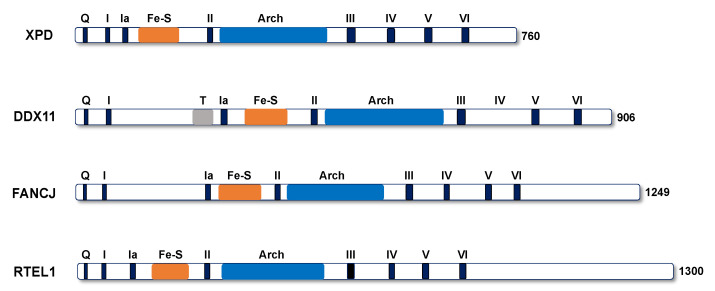
A schematic representation of the polypeptide chain of human super-family 2 DNA helicases containing an Fe–S cluster is reported. Color code is as follows: *blue*, conserved helicase motifs (Q motif, *Q*, and helicase boxes from I to VI); *grey*, Timeless-binding motif (*T*) [12]; *orange*, Fe–S cluster (*Fe–S*) and *pale blue*, Arch domain (*Arch*) domain.

DDX11/ChlR1 (Chl1 in yeast) belongs to the group of super-family 2 (SF2) DNA helicases characterized by the presence of an iron–sulfur cluster (Fe–S) domain [7,8,9]. This subclass also includes the *Xeroderma pigmentosum* group D (XPD) protein, FANCJ, and RTEL1 (see Figure 1). All these DNA helicases are genetically linked to rare hereditary diseases that display genome instability features and, in some cases, also cancer susceptibility. Bi-allelic mutations of the *DDX11* gene cause Warsaw breakage syndrome (WABS), a rare pathology that is characterized by a complex spectrum of symptoms including pre- and post-natal growth defects, microcephaly, various degree of intellectual disability, heart defects, and at cytological level sister chromatid cohesion abnormalities, giving rise to an altered chromosome morphology [10]. The chromosomal cohesion anomalies observed in fibroblasts or lymphoblasts derived from WABS patients or other DDX11-deficient cells consist of a premature centromere division (PCD), characterized by a loosened centromere constriction, likely due to repulsion of the corresponding heterochromatic regions; besides, in an appreciable number of metaphase chromosome spreads of the above cell lines, all the sister chromatid pairs appear completely separated, a condition defined as premature chromatid separation (PCS) [11].

The involvement of DDX11 in chromosomal cohesion is an evolutionarily conserved cellular function of this enzyme, as indicated by the fact that *CHL1*/*CTF1*, the yeast counterpart of *DDX11*, was originally identified in a genetic screening of *Saccharomyces cerevisiae* aimed to identify mutants with reduced chromosome transmission fidelity (CTF) leading to chromosome loss (CHL) [13]. Biochemical characterization of DDX11, initially carried out in the laboratories of Lahti [14] and Hurwitz [15], and then by Brosh and collaborators [8,16,17,18], revealed that the recombinant protein displays an ATPase-dependent DNA unwinding activity with a 5′ to 3′ directionality *in vitro*. DDX11 is optimally active on forked duplex DNA with a 5′-tail of at least 15 nucleotides and a 3′-tail ranging from 5 to 10 nucleotides, while duplex DNA having blunt ends or only a 3′-overhang are not unwound [16]. Besides, in the Brosh laboratory, DDX11 was found to be able to resolve displacement loops but not Holliday junction-containing DNA substrates, suggesting that this activity could be relevant in homologous recombination (HR)-based DNA repair reactions and/or telomere maintenance, because three-stranded displacement loops resemble telomeric-loops (T-loops) that are present at the chromosome ends. Recombinant DDX11 was also able to disrupt high-affinity streptavidin–biotin interaction in a helicase protein concentration- and ATP-dependent manner in assays where biotinylated oligonucleotides bound to streptavidin were used as substrates. This activity is shared by FANCJ among the SF2 Fe–S cluster DNA helicases, but not by some of the RECQ-like DNA helicases (RECQ1, Bloom and Werner helicases) [19]. Nonetheless, it is not completely clear if the ability of these DNA helicases to displace DNA-bound proteins in vitro may have any physiological meaning.

An additional important activity of DDX11 is its ability to dismantle unconventional DNA structures, such as G-quadruplexes (G4) and triplexes, that are believed to arise at genomic sites that have a peculiar sequence composition. G4 structures can form at G-rich sequences due to the propensity of its guanine base stretches to form planar tetrads via Hoogsteen interactions. These tetrads are stabilized by metal ions (especially K^+^) and assembled into stacks [20]. G4 DNA may have a different structure and composition, being formed by four (tetra-molecular) or two (bimolecular) or only one (unimolecular) G-rich strand. Besides, the DNA strands forming a G4 structure can run parallel or anti-parallel or have a mixed arrangement. Although several studies have revealed that G4 structures may impact many genome metabolism pathways, including gene transcription regulation, DNA replication and telomere maintenance, their physiological relevance has been disputed, and only very recently has their existence in live cells been robustly proved using innovative single-molecule real-time imaging technique based on G4-specific fluorescent probe [21,22]. However, it is well known that G4 is an important source of replication stress, especially in highly proliferating cells cancer cells, because they represent a roadblock for the advancing replication machineries. In fact, the replicative DNA helicase, the Cdc45/MCM2-7/GINS (CMG) complex, is unable to dismantle G4 DNA structures. The consequent replication stress originating at G4 sites during S phase is alleviated only thanks to auxiliary DNA helicases, able to efficiently untangle these structures. Many human DNA helicases were demonstrated to be able to resolve G4 DNA structures *in vitro* with different specificity, including RECQ-like and SF2 Fe–S-containing DNA helicases, DNA2 endonuclease/helicase, DHX36, and PIF1 [23,24,25]. DDX11 was shown to resolve only bi-molecular anti-parallel G4 DNA with two 5′-overhangs with high catalytic efficiency, whereas it is only poorly active on parallel tetramolecular G4 DNA and not active at all on unimolecular G4 DNA [16,18]. Conversely, the related FANCJ DNA helicase is able to efficiently resolve all the above G4 DNA structures, especially unimolecular G4s.

Moreover, DDX11 was also reported to dismantle triple-stranded DNA substrates (triplexes) more efficiently compared to other DNA helicases (such as Werner, Bloom, DHX9 and FANCJ) [26,27,28]. DNA triplexes, also named hinge DNA or h-DNA, are formed at poly(purine/pyrimidine)-rich regions of the human genome and derive from non-canonical Hoogsteen hydrogen bonding, as G4 structures. [29,30]. The presence of DNA triplexes in cells was revealed by indirect immunofluorescence experiments with specific antibodies indicating that these peculiar structures may truly form in living cells [31,32].

The physiological relevance of the DDX11 ability to resolve alternative DNA structures is testified by the finding that DDX11-depleted cell lines are extremely sensitive to drugs that specifically bind and stabilize G4 DNA structures. In fact, it has been reported that the G4-stabilizers Quarfloxin and CX-5461 are both very toxic to cells lacking DDX11, but hardly affect *FANCJ*-knockout cells [33]. In contrast, Telomestatin was found to be more harmful to FANCJ- compared to DDX11-deficient cells [18]. The observed differences could be due to the fact that these compounds may target G4s having a different structure/conformation and/or different sub-cellular localization. In fact, Quarfloxin was shown to accumulate in the *nucleolus*, where it inhibits rRNA gene transcription by RNA polymerase I [34]; on other hand, it is known that Telomestatin specifically targets the chromosome telomeric ends, where it inhibits the activity of telomerase [35]. DDX11, which was also localized in the *nucleolus* of interphase cells [36], is able to resolve only bi-molecular anti-parallel G4s with high catalytic efficiency, tetramolecular parallel G4s with poor efficiency, but not unimolecular G4s, as previously mentioned. Of note, studies carried out in the fission yeast *Schizosaccharomyces pombe* indicated that the G4 structures present at the chromosome telomeric ends are mainly unimolecular, whereas intermolecular (bi- or tetra-molecular) G4 structures are prevalent at rRNA gene clusters in the nucleolus [37]. Collectively, all these findings suggest that DDX11 may have an important role in rDNA/rRNA metabolism.

## 3. Cellular Functions of DDX11

In mammalian cells, a pivotal role in counteracting replication stress is played by the so-called fork-protection complex (FPC) that is formed by Tim/Timeless, Tipin and Claspin, three stable components of the DNA replication machinery. The FPC has multiple roles: it is responsible for activating the S phase checkpoint; it has an important, although not yet defined, function in telomere metabolism; and it coordinates DNA synthesis and unwinding by physically bridging the DNA polymerases with the CMG complex at the replication fork [38]. The Pisani group, in collaboration with the Brosh laboratory, demonstrated that DDX11 and Timeless physically and functionally interact and act jointly to preserve replication fork integrity in stressful conditions in HeLa cells [39]. These authors reported that Timeless remarkably stimulates the DDX11 DNA helicase activity in assays carried out *in vitro* with various DNA substrates (forked duplex, displacement loop and G4-containing DNA). More recently, Pisani and collaborators went on to identify a DDX11 sequence responsible for the direct interaction with Timeless, the so-called “E-Y-E” motif, which is invariant in the metazoan DDX11 orthologs and maps in the insertion of the DDX11 polypeptide chain between the conserved helicase boxes I and Ia [12]. This work revealed that the DDX11/Timeless interaction is also critical for recruiting DDX11 and the cohesin complex to the ongoing replication forks and promoting sister chromatid cohesion establishment. In line with these findings, it has been demonstrated that DDX11 and Timeless operate in the same pathway that preserves epigenetic mark inheritance at the *BU-1* chromosomal locus in chicken DT40 cells [39]. The authors of this study have proposed that DDX11, in combination with Timeless, resolves a stable G4 structure located in front of the *BU-1* gene promoter, allowing the replication machinery pass smoothly through this site. Interestingly, in this study, Timeless has been demonstrated to bind various types of G4s with high affinity through a conserved C-terminal α-helical domain. Of note, in the DT40 cell system, co-depletion of both DDX11 and FANCJ has an additive effect on the loss of the epigenetic marks at the *BU-1* site, indicating that these two DNA helicases might operate in independent parallel pathways important for G4 resolution at the replication fork [40]. In line with these results, in a very recent paper by the laboratory of Job de Lange and Rob Wolthuis on novel WABS cases, it has been reported that loss of both DDX11 and FANCJ has additive effects in DNA damage accumulation in various human cell lines following treatment with Pyridostatin, a G4-stabilizer, or mitomycin C, a DNA cross-linker [33]. This again suggests that DDX11 and FANCJ have redundant functions in resolving problems occurring at the replication forks, as previously mentioned.

The molecular mechanism by which DDX11 and its yeast ortholog Chl1 promote sister chromatid cohesion establishment at the replication forks has not yet been fully elucidated. One prevailing hypothesis is that DDX11 resolves G4s and other DNA secondary structures and creates enough “room” on the lagging strand to enable efficient DNA entrapment by the cohesin complex. In fact, helicase-dead variants of DDX11 are unable to rescue sister chromatid cohesion defects of *DDX11*-knockout cell lines in many diverse systems, indicating that the integrity of the DDX11 helicase activity is important for its function in chromosomal cohesion [33,41]. However, a helicase-independent role of Chl1/DDX11 in loading and or anchoring cohesin at the replication forks cannot be completely excluded [12,42].

It was proposed that DDX11 might have a role in heterochromatin organization by recruiting the heterochromatin protein-1, isoform α, (HP1α) at chromosomal pericentric and telomeric sites [43]. This raises the possibility that DDX11 may also have a role in regulating general chromosome architecture and, thus, may influence gene accessibility/transcription. Notably, it was shown that in mammalian cells during interphase, DDX11 is also located in the nucleolus, where it recruits the upstream binding factor (UBF) and a subunit of RNA polymerase I (named RPA194) to the promoter of the 47S ribosomal RNA gene. In fact, *DDX11*-knockdown was found to reduce recruitment of the transcriptional machinery at rRNA gene promoters, suppressing rRNA transcription and inhibiting proliferation of HeLa cells [36].

## 4. Regulation of DDX11 Function in Sister Chromatid Cohesion by the lncRNA *CONCR*

**Figure 2:**
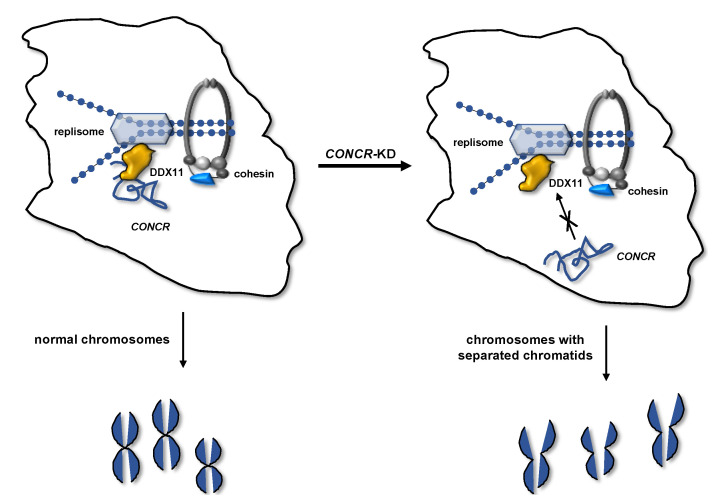
Regulation of sister chromatid cohesion by the lncRNA *CONCR*. *CONCR* transcription is directly activated by Myc in a p53-deficient background. *CONCR* enhances the DDX11 catalytic activity, promoting sister chromatid cohesion establishment at the replication forks. *CONCR* knockdown causes sister chromatid cohesion loss.

A report by Huarte and collaborators revealed the important role played by a long noncoding RNA (lncRNA) in regulating sister chromatid cohesion and DDX11 activity [44]. These authors performed a search for lncRNAs having an altered expression in HCT116 colon carcinoma cells that had a non-functional p53 background. Among the 81 identified lncRNAs, whose expression was found to be enhanced in these cells, they decided to focus on one that was previously annotated as *DDX11 antisense RNA 1* (*DDX11-AS1*; see Section 7). They found that this lncRNAS, renamed “cohesion regulator noncoding RNA” or *CONCR*, is directly regulated by Myc. This was described to be transcriptionally repressed in a p53-dependent manner; therefore, it can be inferred that *CONCR* expression is regulated by p53 in an indirect manner through Myc. Thus, *CONCR* level of expression was found to be higher in p53-deficient compared to p53-proficient cancers. Then, Huarte and collaborators analyzed the ability of *CONCR*-depleted cells to form tumors after injection into nude mice, demonstrating that this lncRNA efficiently promotes tumor growth. DDX11 DNA helicase has a well-established role in sister chromatid cohesion; therefore, the authors decided to examine if *CONCR* could be involved in this process. They demonstrated that *CONCR* knockdown caused severe chromosomal cohesion defects, which could be rescued by co-depletion of Wapl, a component of the cohesin-releasing complex. Besides, they found that the DDX11 ATPase activity was enhanced *in vitro* in the presence of *CONCR* compared to control assays containing an unrelated RNA. This was the first evidence that a lncRNA has a role in the sister chromatid cohesion process (see Figure 2). As previously mentioned, *CONCR* is upregulated in many tumors and its downregulation reduces the tumorigenicity of cells in xenograft cancer mouse models, therefore Huarte and collaborators speculated that *CONCR* may exert its pro-tumorigenic function even by regulating the DDX11 catalytic activities [44].

## 5. DDX11 and Human Papillomavirus Replication and Maintenance

**Figure 3:**
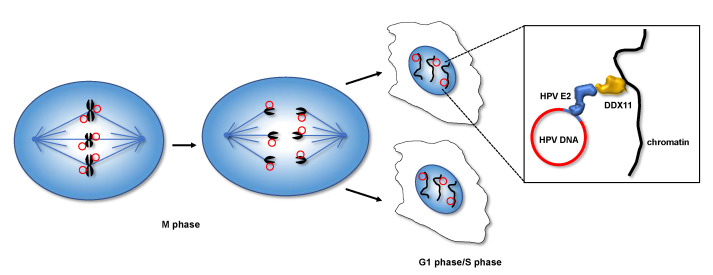
Human papillomavirus (HPV) genome replication and maintenance in infected host cells. The episomal viral DNA molecules (red circles) are tethered to the host cell chromatin throughout the cell cycle. This mechanism enables an even distribution of the viral genomes between the daughter cells, thus ensuring a persistent viral infection. In the inset, the protein bridge made of HPV E2 bound to DDX11 needed for tethering viral DNA to host cell chromatin during S phase is schematically depicted (see text for details).

Human papillomaviruses (HPVs) are double-stranded DNA viruses that infect cutaneous and mucosal epithelia, causing hyperproliferative lesions which are at risk of progressing to anogenital, oropharyngeal, and cutaneous cancers. The papillomavirus life cycle is intimately connected to the epithelial cell differentiation. Viral infection starts in the basal layer of the epithelium, where the viral genome (of about 8000 base-pairs) is maintained as a multi-copy episomal element that is duplicated during the S phase along with the host cell chromosomes [45]. Risk of cancer onset and progression greatly increases upon viral DNA integration into the host cell genome [46]. HPV genome maintenance during persistent infection relies on a mechanism that requires a direct attachment of episomes to the cellular chromatin during the S phase and subsequent cell division. In fact, papillomaviruses do not harbor any centromeric sequence in their genome and do not encode any kinetochore-like structure. For HPV genome to be replicated and transmitted, the role played by the E2 factor, a viral protein important throughout the HPV life cycle, is critical (see Figure 3).

In fact, E2 regulates either papillomavirus genome duplication, interacting with the viral DNA helicase (the E1 protein) or early gene transcription. During differentiation, HPV-infected cells migrate from the basal into the upper layer of the epidermis. At the same time, viral late gene expression is activated, producing structural proteins that eventually assemble into virions. The E2 protein (molecular weight: 42 kDa), present in all papillomaviruses, contains a C-terminal DNA-binding domain (DBD) and a transactivation domain (TAD), which are linked by a flexible hinge region. The E2 DBD specifically recognizes and binds the consensus motif (AACCG(N)_4_CGGTT), which is repeated multiple times in the noncoding regulatory region, named long control region (*LCR*), of the viral genome [47]. The association of E2 with binding motifs located in the *LCR* is necessary for the recruitment of the E1 DNA helicase. This latter, in turn, melts the origin of replication and recruits cellular proteins that are required for replication, such as the replication protein A and the DNA polymerase α-primase complex. At the same time, E2 associates to chromatin-bound proteins of the host cell through its TAD to enable viral genome segregation in the subsequent mitosis. Joanna Parish in the Androphy laboratory demonstrated that the HPV E2 protein interacts with DDX11 using a yeast two-hybrid screening approach; the interaction is mediated by the E2 TAD; the bovine papillomavirus (BPV) E2 protein also binds DDX11 [48]. Additionally, Parish and collaborators found that a BPV E2 variant, which has the amino acid change Trp130Arg in the TAD, displayed remarkably reduced DDX11 binding in co-immunoprecipitation experiments carried out in tumoral C33a cells, transiently co-transfected with plasmids expressing the two proteins. Of note, this papillomavirus was found to be unable to maintain viral episomal elements at a high copy number, even if it retained E2-dependent transcriptional activation and replication functions. These findings revealed the importance of the E2–DDX11 interaction for papillomavirus infection persistence and were also confirmed for human papillomavirus in a study where the Tyr131Ala mutant of HPV E2 protein was found to have a reduced ability to bind DDX11, as the corresponding BPV E2 Trp130Arg variant [49]. Besides, indirect immunofluorescence studies in synchronized cells revealed that E2 and DDX11 co-localize on condensed chromatin during pro-metaphases; although in subsequent steps, DDX11 appeared at the spindle poles, whereas E2 remained on chromosomes until the completion of mitosis. Thus, it was proposed that DDX11 is important for recruiting papillomavirus genomes to the host chromosomes only in S phase but not throughout mitosis [48]. This was confirmed by live imaging studies performed in h*TERT*-immortalized retinal pigment epithelial (h*TERT*-RPE1) cells that were transiently transfected with plasmids expressing E2 fused to the cyan fluorescent protein (E2–CFP) and DDX11 fused to the yellow fluorescent protein (DDX11–YFP) [50]. Then, the dynamic *in-vivo* association of E2–CFP and DDX11–YFP was monitored by fluorescence resonance energy transfer (FRET) measurements in synchronized cells, indicating that the two fluorescent-labelled proteins are in close proximity only from G1 until mid-S phase, but not in mitosis, confirming previous experimental observations [48].

It is noteworthy that the region of the DDX11 polypeptide chain involved in E2 binding encompasses residues 65–225 and corresponds to the N-terminal insertion between helicase box I and Ia, uniquely found in FANCJ and DDX11 among the Fe–S cluster SF2 DNA helicases [9] (see Figure 1). This insertion also contains the Glu200-Tyr201-Glu202 (E-Y-E) Timeless-binding peptide that is invariant in the metazoan DDX11 orthologs [12]. All that considered, it would be interesting to analyze if there was any overlap between the E2- and Timeless-binding sites of DDX11 and if interaction of E2 and Timeless to DDX11 is mutually exclusive. Of note, Timeless was demonstrated to be required for stable episomal maintenance of the duplex DNA genome of human Epstein–Barr virus (EBV) and Kaposi’s sarcoma-associated herpesvirus (KSHV) [51,52]. It should be pointed out that both DDX11 and Timeless are sister chromatid cohesion establishment factors that act jointly at the replication forks to promote the stable tethering of newly duplicated DNA molecules by the cohesin rings. Thus, it is tempting to predict that cohesin and its regulatory network may have a role in pairing the duplicated papillomavirus genomes to permit their subsequent even distribution to the daughter cell nuclei. However, although a direct interaction between the papillomavirus E2 protein and DDX11 has not yet been formally proven and an involvement of Timeless and/or cohesin in HPV genome maintenance is only speculative, elucidating the molecular mechanism by which viral DNA is replicated and transmitted in the infected mammalian cells will not only help better understand the cellular chromosome segregation process, but also provide a useful clinical target for novel antiviral therapeutic approaches.

## 6. Is DDX11 an Oncogene?

Genome sequencing projects have revealed that the *DDX11* gene is amplified and/or mutated in many tumors. Besides, immuno-histochemistry analyses have shown that the DDX11 protein is over-expressed in many cancerous tissues compared to neighboring healthy counterparts, such as in hepatocellular carcinomas, osteosarcoma, melanoma and lung adenocarcinoma. In addition, poor overall survival rate has been usually described for probands affected by malignancies where DDX11 is upregulated. These studies, which are described in more detail in the next subsections, would indicate DDX11 being oncogenic more than a tumor suppressor function, at least in certain cancer types.

### 6.1. DDX11 in Hepatocellular Carcinoma

Hepatocellular carcinoma (HCC) is one of the six most common cancers in the world, and causes approximately 600,000 deaths every year, being the third most lethal tumor type. Chronic hepatitis B/C viral infection is an important cause of HCC. Patients are often diagnosed when the disease is at an advanced stage and surgical intervention is not possible. Additionally, high recurrence rate and poor prognosis are due to frequent intrahepatic and extrahepatic metastasis in HCC patients [53]. All that considered, there is an urgent need of novel diagnostic and prognostic markers and therapies for this tumor. Two very recent studies have revealed the potential involvement of DDX11 in HCC onset and progression [54,55]. Firstly, both these articles have reported compelling evidence that DDX11 expression is remarkably increased in tumoral tissue samples compared to the nontumoral counterparts. In addition, *DDX11* upregulation has been found to not be associated with alcohol consumption and hepatitis infection, although both have been demonstrated to be important HCC risk factors. In fact, cirrhotic tissues, adjacent to HCC, do not display elevated level of DDX11. Besides, in both studies, the existence of a direct correlation between increased DDX11 expression and reduced survival of HCC patients has been highlighted. In fact, *DDX11* gene downregulation has been found to suppress proliferation and colony formation of a number of HCC cell lines (such as HepG2, SMMC7721 and PLC8024). Additionally, when SMCC7721 cells, where DDX11 is depleted or overexpressed, were inoculated into nude mice, liver tumor growth was inhibited or stimulated, respectively, suggesting that DDX11 has a pro-tumorigenic role in these xenograft cancer animal models [54]. To define the molecular mechanisms underlying the oncogenic activity of DDX11 in HCC, Yu and collaborators focused on the PI3K/AKT/mTOR pathway, based on the results of a bioinformatics analysis (gene set variation analysis, GSVA), indicating that genes of this pathway are altogether considerably enriched in HCC tissues displaying high DDX11 expression. Consistently, it has been found that DDX11-downregulation or -overexpression reduces or promotes the phosphorylation of PI3K, AKT and mTOR, respectively. These results indicate that DDX11 supports HCC progression, at least in part, through activating the PI3K/AKT/mTOR signaling pathway [54]. Nonetheless, these authors have not elucidated the mechanism by which DDX11 promotes activation of the above signaling pathway that has an important influence on cell proliferation and on the growth and progression of many tumors.

**Figure 4:**
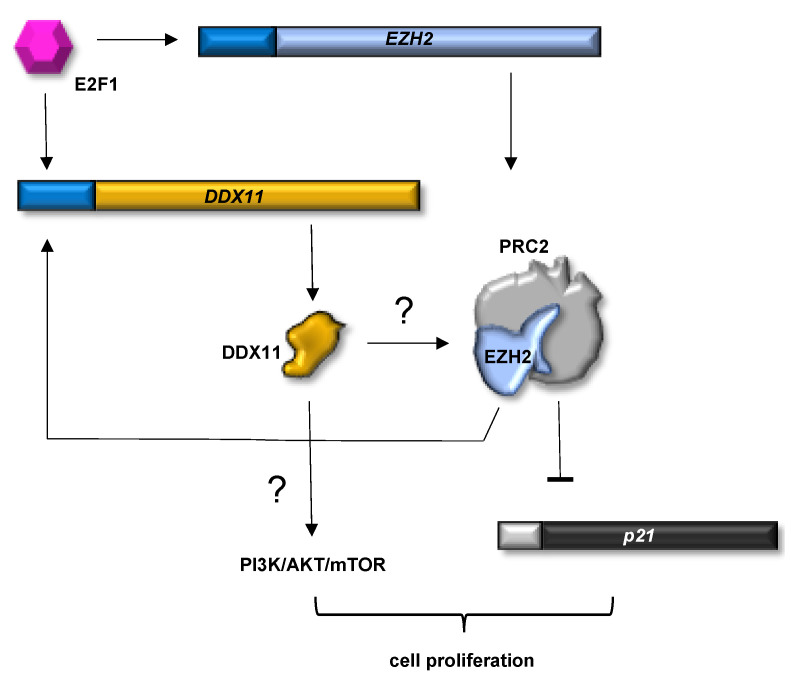
DDX11-mediated regulation of EZH2-p21 and PI3K/AKT/mTOR signaling pathways in HCC cells. The E2F1 factor promotes transcription of the genes coding for DDX11 and the Enhancer of zeste homolog 2 (EZH2), the subunit with methyl-transferase activity of the polycomb repressive complex 2 (PRC2). DDX11 has been proposed to activate the PI3K/AKT/mTOR signaling pathway and stabilize EZH2 by preventing its proteolytic degradation (question marks indicate that the underlying molecular mechanisms have not been elucidated). In turn, stabilized EZH2 cooperates with E2F1 to activate *DDX11* gene transcription forming a positive feedback loop that leads to cell proliferation. Besides, inhibition of *p21* gene transcription by EZH2 also contributes to cell growth.

Another important finding of both these studies is that *DDX11* gene transcription is under the control of the E2F1 factor in HCC cell lines; E2F1 loss causes a reduction in *DDX11* mRNA levels, while E2F1 over-expression enhances DDX11 protein levels (see Figure 4).

Additionally, the authors have found that E2F1 directly binds to the *DDX11* promoter region by chromatin immunoprecipitation (ChIP) experiments, and in HCC cells, where *DDX11* is upregulated, level of the p21 tumor suppressor is reduced, whereas *DDX11*-knockout restores p21 expression [54,55]. However, in the paper by Su and collaborators, DDX11 has been found to associate in cells with the Enhancer of zeste homolog 2 (EZH2), the catalytic subunit of the polycomb repressive complex 2 (PRC2). EZH2 tri-methylates Lys-27 of histone 3 (H3K27me3) at target gene promoters, altering their downstream expression [56]. Levels of ubiquitinated EZH2 are increased in DDX11-depleted HCC cells; therefore, it has been proposed that DDX11 might protect EZH2 from proteasome-mediated degradation in these cell lines inhibiting its ubiquitination. However, it is not clear from this study how DDX11 would prevent EZH2 ubiquitination and subsequent degradation, and no evidence that the two proteins may establish a direct interaction has been reported to clarify the underlying mechanism. It is known that the *p21* gene is epigenetically downregulated by EZH2 in a p53-independent manner [57]; therefore, it has been proposed that DDX11 might indirectly suppress *p21* transcription by enhancing the EZH2 protein stability [55]. Moreover, because E2F1 activates *EZH2* transcription and, in turn, EZH2 cooperates with the E2F1 to promote *DDX11* transcription, existence in HCC cells of a positive feedback loop on the E2F1/DDX11/EZH2 axis has been hypothesized. Collectively, the results of these studies reveal that DDX11 exerts a pro-tumorigenic activity *via* PI3K/AKT/mTOR [54] and E2F/EZH2/p21 [55] signaling pathways in HCC (see Figure 4) and suggest that DDX11 can be a novel prognostic biomarker for this cancer type.

### 6.2. Role of DDX11 in Osteosarcoma

Osteosarcoma is an aggressive malignant neoplasm that derives from transformed cells of mesenchymal origin undergoing osteoblastic differentiation with formation of a malignant osteoid. This kind of bone tumor is most prevalent in teenagers and young adults and displays high mortality. Surgical intervention combined with chemotherapy is the only current treatment available for osteosarcoma [58]. Interestingly, one study aimed to explore and understand the mechanisms underlying osteosarcoma onset and progression and has revealed the important role played by the long noncoding (lnc) RNA named *DDX11 antisense RNA 1* (*DDX11-AS1*) [59]. LncRNAs are transcripts with a length of more than 200 nucleotides which are synthesized by the RNA polymerase II complex; they do not contain an open reading frame but have mRNA-like characteristics (5′-cap and 3′-polyA). LncRNAs represent more than 25% of all human genes (GENCODE v24), but only a small number of them have been functionally characterized to date. LncRNAs may regulate multiple important cellular pathways (cell cycle progression, nuclear transport, gene transcription, chromatin remodeling, and others) and their dis-regulated expression can lead to tumor development [60,61,62,63]. *DDX11-AS1* was discovered several years ago as a lncRNA upregulated in hepatocellular carcinoma (HCC) [64]. *DDX11-AS1* is transcribed from the site that harbors the *DDX11* gene on chromosome 12 (*locus 12p11.21*), but in the opposite direction and without overlap with the *DDX11* mRNA [65]. Zhang and collaborators have found that *DDX11-AS1* is remarkably over-expressed in osteosarcoma cell lines (especially U2OS and HOS) and its downregulation restrains cell proliferation, migration, and invasion [59]. Besides, in *DDX11-AS1*-depleted cells, a reduced expression of epithelial–mesenchymal transition (EMT) markers (such as MMP2, MMP9, N-cadherin, Slug and Twist) has been observed, suggesting that loss of this lncRNA may suppress the EMT process. Additionally, these authors have found that levels of *DDX11* mRNA and DDX11 protein are reduced in U2OS and HOS cells when *DDX11-AS1* is downregulated. They have found that *DDX11-AS1* specifically binds *miR-873-5p* in U2OS and HOS cell extracts, as indicated by the results of pulldown experiments using a *DDX11-AS1* biotinylated probe. *miR-873-5p* is a microRNA reported to be important for regulating EMT in various cancer types [66]. It is considered a tumor suppressor because it inhibits proliferation, migration, and invasiveness of U2OS and HOS cells when upregulated. Zhang and collaborators have reported that *DDX11-AS1*, *miR-873-5p* and *DDX11* mRNA are all associated with the RNA-induced silencing complex (RISC), as indicated by RNA immunoprecipitation (RIP) experiments with an anti-Argonaute protein antibody in osteosarcoma cell lines. Based on these results, the authors have proposed that *DDX11-AS1* might “sponge” *miR-873-5p*, suppressing its ability to downregulate DDX11 expression in osteosarcoma cells [59] by a well-known regulatory mechanism described for other microRNAs [67]. Moreover, in the same study, the authors have shown that *DDX11-AS1* is specifically bound by the insulin-like growth factor 2 mRNA-binding protein 2 (IGF2BP2), an RNA-binding protein that plays an oncogenic role in many cancers [68,69]. Downregulation of IGF2BP2 reduced *DDX11* mRNA stability and DDX11 protein production in U20S and HOS cells. Of note, in the same study it was found that DDX11 overexpression could reverse the inhibition of proliferation, migration and invasiveness of U2OS and HOS cells due to *DDX11-AS1* loss and, consistently, experiments in xenograft nude mice osteosarcoma model have revealed that DDX11 over-expression could countervail the suppressive role of silenced *DDX11-AS1* on tumor growth. In conclusion, this study has revealed that *DDX11-AS1* regulates *DDX11* gene expression in osteosarcoma cells with a dual mechanism: on one hand it sequesters *miR-873-5p* preventing it from silencing the *DDX11* mRNA through RISC; on the other hand, it associates with the IGF2BP2 protein factor supporting its stabilizing effect on *DDX11* mRNA [59]. The results of this study indicate that *DDX11-AS1* lncRNA and the DDX11 DNA helicase can be considered as novel targets in therapeutic strategies for treating osteosarcoma.

### 6.3. Role of DDX11 in Lung Adenocarcinoma and Melanoma

Lung cancer is the most common cause of cancer-related death in men, and second most common in women after breast cancer. Most cancers that start in the lung, known as primary lung cancers, are carcinoma. The two main types are small-cell lung carcinoma (SCLC) and non-small-cell lung carcinoma (NSCLC). This latter is more frequent, accounting for about 80–85% of all lung cancers, with lung adenocarcinoma (ADC) being its most common subtype [70]. Despite the great advances made in the diagnosis and therapy of ADC, the five-year overall survival of ADC patients is approximately 50–70% due to the features of high metastasis and recurrence displayed by this malignancy.

Database analysis has revealed that *DDX11* mRNA is upregulated in many ADC samples compared to healthy tissues. [71,72]. DDX11 over-expression has been found to be significantly correlated with decreased overall survival rates for ADC. In addition, immunohistochemistry studies carried out on tissue samples derived from an ADC patient cohort have revealed a positive correlation between DDX11 levels of expression and tumor size and stage. Univariate and multivariate analyses have suggested that DDX11 can be an independent prognostic marker for ADC patients.

Melanoma is the most life-threatening common form of skin cancer. In early stages, melanoma treatment includes surgery alone and the survival rates are high. However, after metastasis, survival rates drop significantly. Therefore, early diagnosis is a crucial factor in treating this type of cancer for which two therapies have been devised so far: one based on BRAF small molecule inhibitors, and the other one on monoclonal antibodies specific for cytotoxic T lymphocyte–associated antigen 4 (CTLA-4). Nonetheless, resistance to BRAF inhibitors and limited efficacy of the immunotherapy has prompted researchers to keep identifying novel therapeutic targets for this aggressive cancer [73]. Research published by the Becker group reported the identification of *DDX11* as a gene that is upregulated eight-fold in radial growth phase (RGP) melanoma, the first stage of invasive melanoma, compared to non-invasive melanoma in situ (MIS) [74]. This analysis was carried out on RNA extracted from tumor tissues samples using whole genome chip arrays. Moreover, immunohistochemistry studies confirmed that the DDX11 expression level is much higher in late-stage melanoma and melanoma-infiltrated lymph nodes compared to normal melanocytes, atypical nevocytes and MIS-derived tissues. Then, the same authors analyzed the phenotypic consequences of silencing *DDX11* with siRNAs in cell lines derived from metastatic growth phase melanoma and found evident morphological alterations, sister chromatid cohesion anomalies, chromosome breakages, telomere shortening, cell proliferation inhibition, and massive apoptosis. Based on these results, Becker and colleagues proposed that DDX11 could be considered as a novel target in alternative strategies to cure advanced melanoma that are resistant to other pharmacological treatment or refractory to radiation therapy [73,74].

## 7. Conclusions

In a review article published in 2015 by Pearl and collaborators about the identification of DNA damage response proteins that are deregulated in various cancers and which could represent novel important therapeutic targets, DDX11 was mentioned as a potential oncogene [75]. This classification relies on the “20:20 rule” proposed to discriminate tumor suppressor genes (TSGs) from oncogenes (OGs) based on their mutational pattern across tumor samples. According to this rule, a gene that has >20% truncating/inactivating mutations in cancer tissues can be considered a TSG; however, if a gene has >20% missense mutations in recurrent positions, it can be considered as an OG [76]. The prediction about a putative tumorigenic role of DDX11 appears to be confirmed by contemporary reports indicating that *DDX11* over-expression promotes hepatocellular carcinoma and osteosarcoma growth and metastasis, while *DDX11* downregulation restrains tumoral development and progression in xenograft mouse models of these cancers, as we have summarized herein. Nonetheless, as observed for other genome stability maintenance DNA helicases [1,3], it is plausible that *DDX11* may have a role as a tumor suppressor gene in different cellular backgrounds, consistently with the finding that its hypomorph and loss-of-function mutations lead to sister chromatid cohesion defects, chromosomal breakage, and genome instability [33]. Besides, it should be also considered that *DDX11* upregulation mediated by E2F1 is not surprising, because E2F transcription factor family members were reported to promote the expression of many genes needed during S phase, including a number of DNA replication factors. In addition, in many cancer types, where E2F transcription factors are activated, due to retinoblastoma (Rb) protein mutations and/or amplification of *Cyclin D* or *Cyclin E* genes, all the relevant downstream target genes are found to be upregulated.

Collectively, these findings suggest that DDX11 can be a novel prognostic biomarker for those tumors where it is over-expressed. However, evidence that *DDX11* behaves as an oncogene in these tumors is not definitive and additional studies are needed to reinforce this proposal. Nevertheless, the discovery of small molecule compounds that specifically inhibit DDX11 catalytic activities is welcome, because it may pave the way to the design of novel anti-cancer pharmacological treatments.

## Data Availability

Not applicable.
